# Acrolein production from glycerol dehydration over amorphous V–P–N–C catalysts†

**DOI:** 10.1039/d4ra08613a

**Published:** 2025-04-01

**Authors:** Jun Liu, Xiaobing Zhao, Weichen Wang, Youjun Yan, Guofu Huang, Meng Liang, Xinzhen Feng, Weijie Ji

**Affiliations:** a Peninsula Engineering Research Center of Comprehensive Brine Utilization, Weifang University of Science and Technology Weifang 262700 China; b Key Laboratory of Mesoscopic Chemistry, MOE, School of Chemistry and Chemical Engineering, Nanjing University Nanjing 210023 China fxz@nju.edu.cn

## Abstract

Amorphous catalysts exhibit a plethora of oxygen vacancies, electron-rich active sites, and highly dispersed active centers, thereby yielding exceptional catalytic performance for multiple reactions. In this work, a series of amorphous V–P–N–C catalysts were synthesized using complexants and employed for catalyzing the glycerol dehydration reaction towards acrolein. Under optimized reaction conditions, the glycerol conversion reached 99.1% with an acrolein selectivity of 83.2% over the amorphous catalyst VPOC_6_. The comprehensive characterization results of Raman, XPS, H_2_-TPR, SEM, BET, and NH_3_-TPD, demonstrated that the addition and decomposition of 1,6-diaminohexane leads to a transition from crystalline to amorphous state while preserving the fundamental vanadium–phosphorus oxide phases. It results in an active graphite-type nitrogen structure and an abundance of oxygen vacancies, which promote the target reaction by virtue of numerous medium acid sites on the catalyst surface.

## Abstract 2

A highly efficient amorphous V–P–N–C catalyst with a porous honeycomb structure is utilized for the continuous production of acrolein through glycerol dehydration.

## Graphical abstract

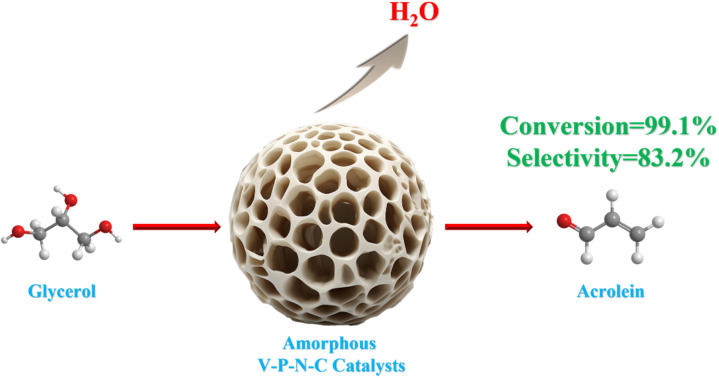

## Introduction

Almost a century on, fossil fuels have contributed significantly to global industrial and economic development. However, the resulting CO_2_ emissions have exerted significant environmental pressure.^1–4^ The ratification of the Paris Agreement and the establishment of CO_2_ reduction targets have generated significant academic and industrial interest in substituting fossil fuels with renewable biodiesel.^5–7^ Transesterification currently represents the predominant method for biodiesel production, leading to the generation of approximately 10% glycerol as a by-product.^8–10^ Consequently, converting surplus glycerol into high-value-added chemicals such as 1,2-propylene glycol,^11^ 1,3-propylene glycol,^12^ glycerol carbonate,^13^ and acrolein^14^ is of significant environmental and economic importance. Particularly in demand within absorbent resins, detergents, fragrances, fuels, and pesticide industries,^15^ acrolein serves as a valuable chemical raw material. Therefore, the dehydration process that converts glycerol to acrolein offers a more cost-effective and sustainable alternative to current petroleum-based production routes.^16^

As a typical acid-catalyzed reaction, glycerol can undergo partial conversion to the target product acrolein on catalysts possessing both Brønsted and Lewis acid sites, such as heteropolyacids,^17^ zeolites,^18^ metal oxides,^1,19–21^ phosphates,^9^ and sulfates.^22^ For instance, Shan *et al.*^5^ employed phosphoric acid impregnation to modify HZSM-5, resulting in complete conversion of glycerol and a selectivity of 89.6% for the target product. Xie *et al.*^1^ employed WO_3_/ZrO_2_@SiC as a microwave-absorbing catalyst to facilitate the target reaction with the assistance of microwaves, achieving a selectivity of over 70% for the target product at 250 °C. Liu *et al.*^14^ prepared an unsupported MoP catalyst using the temperature-programmed reduction method to catalyze the target reaction, remarkably achieving a consistent selectivity of over 80% towards acrolein with a duration of 50 hours. Wang *et al.*^7^ employed tungsten-based heteropoly acid supported on silica as the catalyst and revealed a positive correlation between selectivity and the surface density of the acidic sites.

However, all of the mentioned catalysts are crystalline. According to reports,^23^ crystalline catalysts exhibit a regular and stable structure, as well as commendable catalytic performance. However, the nearly flawless surface usual restricts the presence of oxygen vacancies and active sites crucial for heterogeneous catalytic reactions. The amorphous catalysts, in contrast, display a multitude of defects and oxygen vacancies on surface as a result of the presence of dangling bonds and unsaturated atomic coordination. It provides electron-rich active sites and highly dispersed active centers similar to homogeneous catalysts.^24^ Additionally, the flexible local structure of amorphous catalysts also enables efficient electron transfer during catalytic reactions.^25^ Although there have been relevant reports on the utilization of amorphous catalysts in ozonolysis,^23^ CO_2_ hydrogenation to formic acid,^24^ OER,^25^ cyclohexanol oxidation,^26^ and butane oxidation,^27^ no studies have investigated the application of amorphous catalysts in glycerol dehydration towards acrolein.

Therefore, in this study, a series of amorphous V–P–N–C catalysts were synthesized using complexants and employed for catalyzing the glycerol dehydration reaction towards acrolein. The results demonstrate that amorphous V–P–N–C catalysts, possessing an active graphitized nitrogen structure, endow numerous oxygen vacancies, abundant changeable V species, as well as a large number of medium acidic sites that exhibit catalytic activity superior to that of conventional crystalline vanadium–phosphorus oxide catalysts.

## Experimental

### Materials

The following chemicals, namely ammonium metavanadate (NH_4_VO_3_), phosphoric acid (H_3_PO_4_, 85%), *N*-methylpiperazine (C_5_H_12_N_2_), and 1,6-diaminohexane (NH_2_(CH_2_)_6_NH_2_), are AR in purity. The glycerol is commercially pure.

### Catalyst preparation

In a typical catalyst preparation process, 0.04 mol of NH_4_VO_3_ was added to 90 mL of deionized water and dissolved by stirring at 90 °C to obtain a bright yellow solution. Subsequently, 0.01 mol of complexant was added, and the mixture was refluxed for 1 h. Then a dropwise addition of 0.04 mol of 85% H_3_PO_4_ followed by stirring for 20 min occurred before the water steam distillation. The molar ratio of the coordination agents to vanadium (V) and phosphorus (P) is 1 : 4 : 4. The resulting mixture was dried under vacuum for 24 h to obtain the catalyst precursors named pre-VPOC_5_ and pre-VPOC_6_ using different coordination agents (*N*-methylpiperazine, and 1,6-diaminohexane) respectively. As a control group without complexant, the catalyst precursor was named pre-VPO.

The catalyst precursors obtained (pre-VPOC_5_, pre-VPOC_6_, and pre-VPO) were activated by heating at a rate of 2 °C min^−1^ in an air atmosphere with a flow rate of 60 mL min^−1^ up to 400 °C for 16 h, resulting in activated catalysts named VPOC_5_, VPOC_6_, and VPO, respectively.

### Characterization methods

Various techniques including SEM, BET, Raman, XRD, XPS, H_2_-TPR, and NH_3_-TPD were used to characterize the physical chemical properties of the catalysts. The detailed information was presented in the ESI.†

### Catalyst evaluation

The catalytic activity of all catalysts for the glycerol dehydration reaction towards acrolein was evaluated using an evaluation device under atmospheric pressure. Before evaluation, the catalysts were compressed, crushed, and screened for particles ranging from 20 to 40 mesh. 0.5 g of catalyst was placed in a straight quartz reaction tube with an inner diameter of 10 mm, followed by the addition of quartz sand with the same mesh size to fill the remaining space. The catalyst was preheated in a nitrogen atmosphere at a flow rate of 30 mL min^−1^, with a heating rate of 10 °C min^−1^, until the desired reaction temperature was achieved. It was followed by purging for 2.5 h. Subsequently, 6.0 mL h^−1^ of glycerol solution (equivalent to 27.36 mmol h^−1^ in terms of glycerol) was introduced into the reaction tube using nitrogen and air as carrier gases, with flow rates ranging from 20 to 60 mL min^−1^ and oxygen concentrations varying from 0% to 12%. The temperatures range from 280 to 340 °C. After specific reaction times elapsed, the liquid components were collected and analyzed by cold trap condensation while the tail gas underwent direct analysis using the TCD detection method. Gas chromatography equipped with an FID detector (GC126N) along with an HP-FFAP capillary column (0.32 mm × 0.5 μm × 25 m) was used to analyze the components of the liquid phase after completion.

Formation rate of acrolein (FR_Acr_) is defined by eqn (1):1
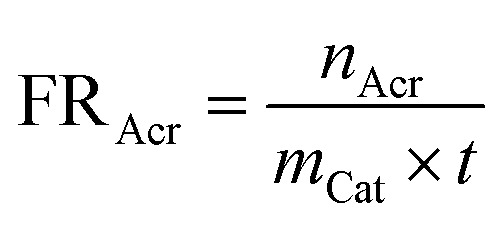
where *n*_Acr_ is the molar quantity of acrolein, *m*_Cat_ is the mass quantity of catalyst, and *t* is the reaction time (150 min).

Selectivity of acrolein (*S*_Acr_) is defined by eqn (2):2
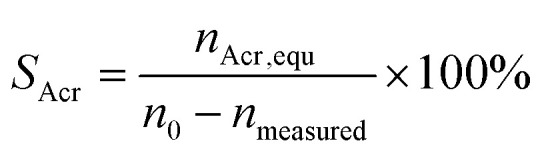
where *n*_Acr,equ_ is the molar quantity of glycerol equivalent to acrolein, *n*_0_ is the molar quantity of glycerol fed into the reactor, *n*_measured_ is the molar quantity of unreacted glycerol.

Conversion of DG (*X*_DG_) is defined by eqn (3):3
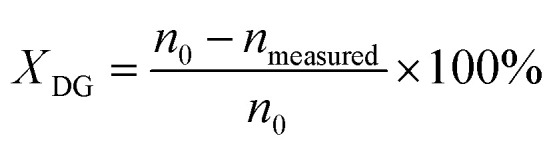
where *n*_0_ is the molar quantity of glycerol fed into the reactor, *n*_measured_ is the molar quantity of unreacted glycerol.

The carbon balance is calculated by eqn (4):4
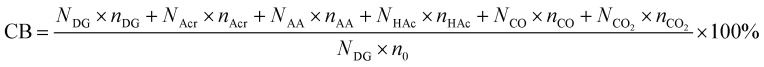
where *N* is the number of carbons in a specific molecule, *n* is the mole quantity of each component measured by GC.

## Results and discussion

### Structure and phase composition

**Fig. 1:**
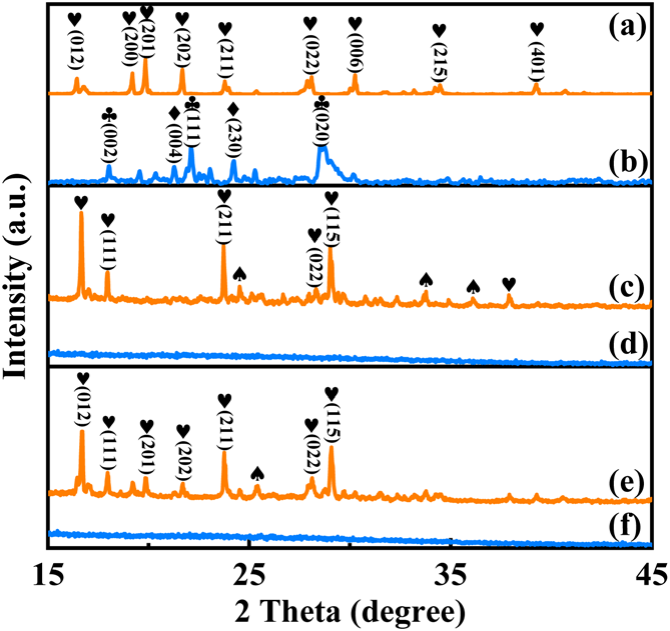
XRD patters of the catalysts, (a) pre-VPO, (b) VPO, (c) pre-VPNC_6_, (d) VPNC_6_, (e) pre-VPNC_5_, and (f) VPNC_5_. ♣-δ-VOPO_4_ PDF # 47-0951, ♦-γ-VOPO_4_ PDF # 47-0950, ♥-(NH_4_)(VO_2_)HPO_4_ PDF # 33-0062, ♠-V–P–N–C coordination compound.

The crystal structures of the precursor and activated catalyst were determined by X-ray diffraction analysis, as shown in Fig. 1. The observed peaks of the catalyst precursor pre-VPO closely match the characteristic diffraction peaks of (NH_4_)(VO_2_)HPO_4_ as reported in the literature,^28^ in the absence of complexant. However, the crystal structure of the precursors pre-VPOC_5_ and pre-VPOC_6_ underwent significant alterations upon the addition of complexants. Besides (NH_4_)(VO_2_)HPO_4_, an extra diffraction peak corresponding to the V–N–C coordination compound was observed,^29^ suggesting that 1,6-diaminohexane and *N*-methylpiperazine were coordinately incorporated into the precursor structure. Furthermore, distinct effects on the crystal orientation of the precursors were observed when different types of complexants were employed, thereby enhancing the structural variability in vanadium–phosphate oxide.^30^ The crystalline of catalyst VPO remains intact in the absence of complexant, exhibiting distinct diffraction peaks corresponding to δ-VOPO_4_ and γ-VOPO_4_ phases, respectively. However, the thermolysis of complexants caused the catalyst to undergo a transition from crystalline to amorphous state by disrupting the hydrogen bond network during the activation process, resulting in structural collapse.

**Fig. 2:**
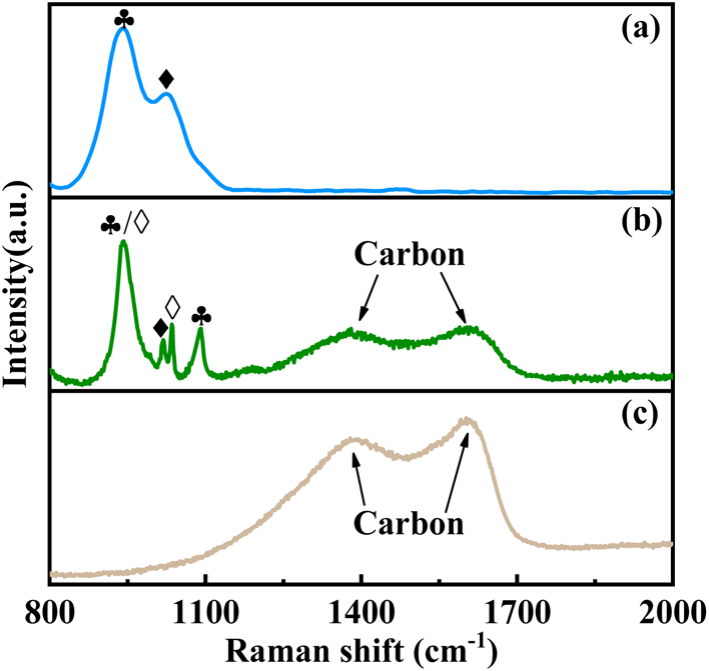
Raman spectra of the catalysts, (a) VPO, (b) VPOC_6_, (c) VPOC_5_. ♣-δ-VOPO_4_, ♦-(VO)_2_P_2_O_7_, ⋄-γ-VOPO_4_.

The utilization of Raman spectroscopy enables a more precise examination of the structure and phase composition of amorphous catalysts, in comparison to XRD.^31,32^ In Raman spectra, vibrational bands corresponding to P–O and V–O bonds typically manifest within the spectral range of 850–1200 cm^−1^, whereas those associated with amorphous carbon are observed across a broad range spanning from 850 to 2000 cm^−1^.^26,33^ As shown in Fig. 2, in absence of complexant, only characteristic Raman bands related to δ-VOPO_4_ (936 cm^−1^) and (VO)_2_P_2_O_7_ (1020 cm^−1^) are present.^31^ However, upon the addition of complexants, distinct Raman bands corresponding to disordered carbon (1350 cm^−1^, D band) and graphitic sp^2^ carbon (1560 cm^−1^, G band) are observed. It is noteworthy that the utilization of *N*-methylpiperazine as a complexant exclusively leads to the appearance of Raman bands associated with carbon, while no bands of P–O or V–O, indicating the degradation of vanadium–phosphorus oxide. However, when 1,6-diaminohexane is employed as the complexant, in addition to the Raman bands of carbon, the bands of vanadium–phosphorus oxides are also observed. And the characteristic band (942 cm^−1^) is altered due to interphase coupling between δ-VOPO_4_ and γ-VOPO_4_, resulting in a beneficial impact on the selectivity of acid–base catalytic reactions.^31^

### BET and morphology

**Fig. 3:**
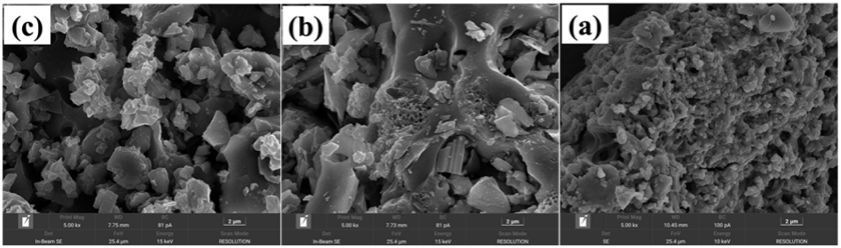
SEM images of the catalysts, (a) VPO, (b) VPOC_6_, (c) VPOC_5_.

As shown in Fig. 3, the results of the SEM demonstrate that the morphology of the catalyst prepared without complexant consists of nanometer-sized particles ranging from 200 to 500 nm. In contrast, catalysts VPOC_5_ and VPOC_6_ exhibit distinct morphological transition from relatively regular nanometer-sized particles to irregular block-like structures. And the catalyst morphology is influenced by the type of complexants employed. The addition and decomposition of *N*-methylpiperazine leads to a significant decrease in the particle size of the bulk catalyst. However, the utilization of 1,6-diaminohexane results in the formation of a porous honeycomb-like structure within the block catalyst, thereby providing an abundance of active sites for catalysis.

**Fig. 4:**
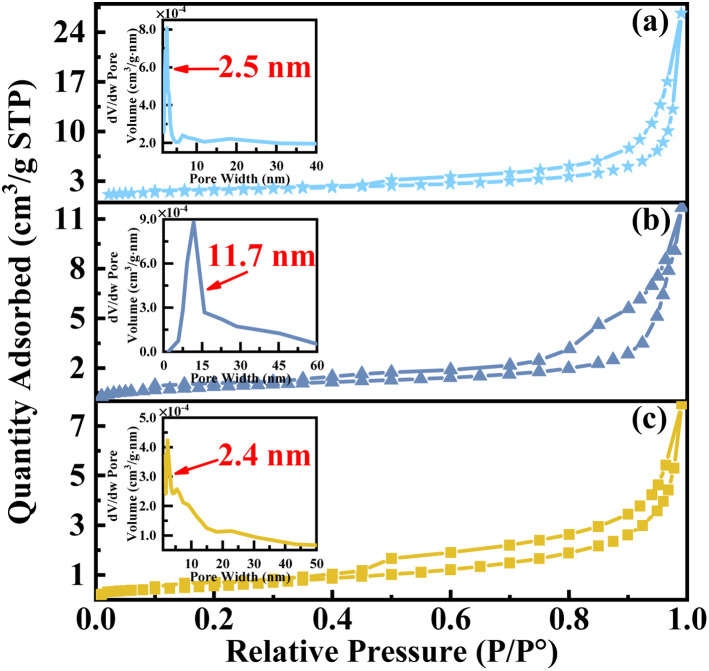
N_2_ adsorption–desorption isotherms of the catalysts, (a) VPO, (b) VPOC_6_, (c) VPOC_5_.

As shown in Fig. 4, the BET results demonstrate that all catalysts exhibit isotherms characterized by a typical IV type N_2_ adsorption–desorption curve, accompanied by an H3 hysteresis loop, indicating the presence of irregular mesoporous structures. Furthermore, the surface area, mean pore volume, and mean pore size (as shown in Table S1†) of the catalysts prepared without complexant exhibit significantly larger values. It can potentially be attributed to an enhanced exposure of crystal faces and pores within the regular crystal structure. However, the pore size distribution curve (insert) indicates that the majority of pores in catalysts VPO and VPOC_5_ are concentrated within the range of 1 to 3 nm. In contrast, utilization of 1,6-diaminohexane as a complexant results in a more focused pore distribution with a predominant range of 11 to 12 nm. Notably, the main pore size distribution of the catalyst is lower than the average pore size, indicating a distinct multistage pore structure. The aforementioned observation is in line with the SEM results and implies that the inclusion of 1,6-diaminohexane as complexant facilitates the formation of a porous honeycomb structure, thereby increasing the number of active sites.

### Surface composition and state

**Fig. 5:**
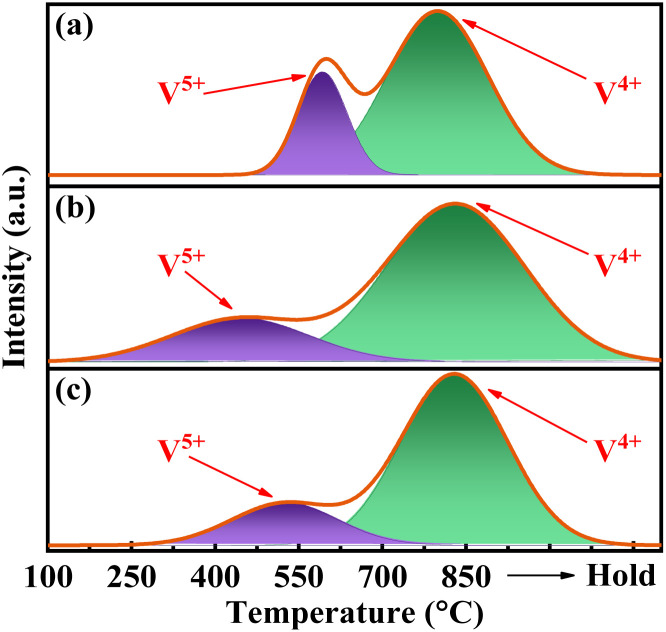
H_2_-TPR profiles of the catalysts, (a) VPO, (b) VPOC_6_, (c) VPOC_5_.

As depicted in Fig. 5, the H_2_-TPR curves for all catalysts exhibit two distinct peaks upon deconvolution. The peak below 700 °C corresponds to the reduction of V^5+^, whereas those above 700 °C correspond to the reduction of V^4+^.^34^ The H_2_ consumption is calibrated accordingly with the utilization of CuO. The results presented in Table S2† demonstrate a significant decrease in the reduction temperature of surface V^5+^ for catalysts VPOC_5_ and VPOC_6_, as compared to those without complexant. Particularly when utilizing 1,6-diaminohexane as complexant, the reduction of surface V^5+^ occurs even at 451 °C. It suggests that the presence of 1,6-diaminohexane leads to an increased density of defects and dangling bonds on the surface of amorphous catalyst, thereby providing more active oxygen sites.

**Fig. 6:**
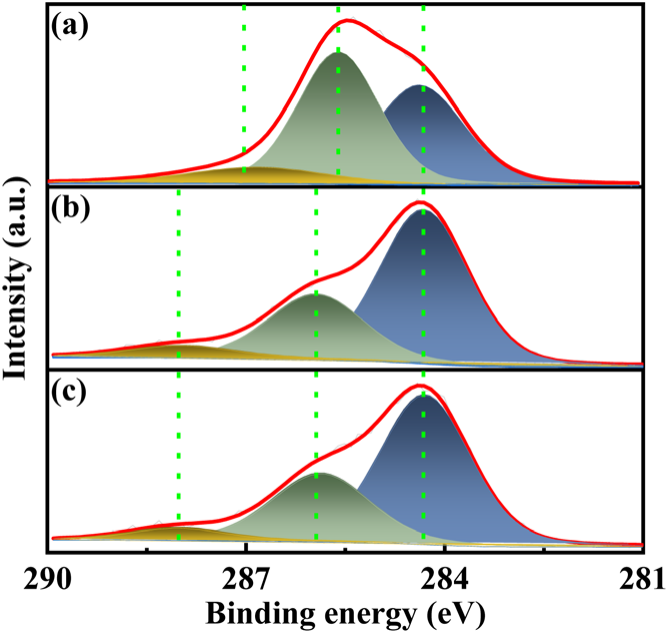
XPS spectra of C 1s for the catalysts, (a) VPO, (b) VPOC_6_, (c) VPOC_5_.

The surface composition was further investigated *via* XPS. As depicted in Fig. 6, the high-resolution C 1s spectra of all catalysts exhibit three distinct peaks. The catalysts VPOC_5_ and VPOC_6_ exhibits distinct C species on surface, including C=C bond (284.3 eV), C–N bond (285.9 eV), and C–O bonds (288 eV).^26^ However, no characteristic signals corresponding to C–O or C–N bonds were detected for the catalyst prepared without complexant. It suggests that the activation process involves chemical interactions between carbon and vanadium–phosphorus oxide, which are induced by the pyrolysis of complexants.

Additionally, significant discrepancies in the surface N species of catalysts were observed upon the utilization with various complexants. As depicted in Fig. S1,† the presence of V–N bonds (397 eV), N–C_3_ bonds (399.7 eV),^26,35^ and graphite-type N (401.8 eV) was clearly observed on the surface of catalyst VPOC_6_. On the contrary, only V–N bonds (397 eV) and C–N=C bonds (398.4 eV) were detected on the catalyst surface when subjected to *N*-methylpiperazine.^26^ It elucidates that during the activation process, pyrolysis transforms the complexant into disordered carbon–nitrogen compounds that interact with vanadium–phosphorus oxide. Obviously, the involvement and pyrolysis of 1,6-hexamethylenediamine specifically contributes to the formation of active graphite-type nitrogen, which plays a crucial role in enhancing catalytic performance.^36^

**Fig. 7:**
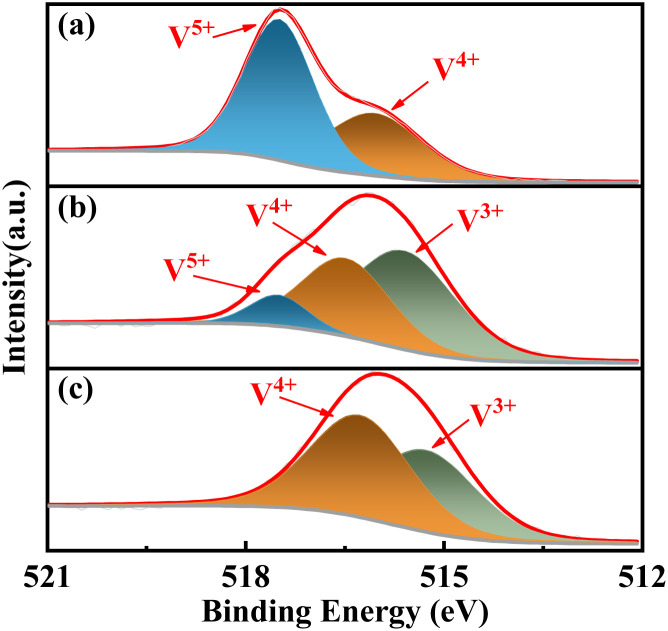
XPS spectra of V 2p_3/2_ for the catalysts, (a) VPO, (b) VPOC_6_, (c) VPOC_5_.

The complexants exerted a significant influence on the surface V and O species of the catalyst. The deconvolution calculation in Fig. 7 revealed that, in the absence of complexant, the V 2p spectra could be separated into two distinct peaks corresponding to V^5+^ (517.5 eV) and V^4+^ (516 eV), with a considerably higher proportion of V^5+^ compared to V^4+^ (Table S3†). It suggests that the reduction degree of V^5+^ is low in when no complexant is employed. However, the addition of complexant resulted in a significant increase in the presence of V^4+^, with even lower valence states such as V^3+^ (515.5 eV) being observed. It indicates that the complexant effectively promotes the reduction degree of V^5+^. Notably, the utilization of 1,6-diaminohexane as complexant results in the simultaneous presence of three valence states, namely V^5+^, V^4+^, and V^3+^. The multivalent system involving V^5+^–V^4+^–V^3+^ may exhibit superior catalytic efficacy for target reactions.

As shown in Fig. S2,† the analysis of O species on the catalyst surface demonstrates that the O 1s spectra can be deconvoluted into three distinct peaks, corresponding respectively to lattice oxygen (O_L_, 530 eV), oxygen vacancy (O_V_, 531.4 eV), and adsorbed oxygen (O_C_, 532.4 eV).^37,38^ The presence of oxygen vacancies (O_V_) has been previously reported to enhance the formation of an electron-depletion layer on the surface,^37^ thereby facilitating REDOX reactions. The results presented in Table 1 demonstrate a significantly higher proportion of O_V_ for catalysts VPOC_5_ and VPOC_6_. It suggests that the inclusion and pyrolysis of complexants lead to a transformation from crystalline to amorphous state, thereby promoting the formation of O_V_. The presence of O_V_ has been reported to enhance the density of Lewis acidic sites on the catalyst surface by increasing the number of surface hydroxyl groups, thereby improving catalytic performance.^39^ It also provides an explanation for the superior catalytic activity observed in catalyst VPOC_6_, which possesses the highest number of O_V_.

**Table 1:** XPS results of O 1s for the catalysts

Catalysts	O_L_ (%)	O_V_ (%)	O_C_ (%)
VPO	73.1	20.5	6.4
VPOC_6_	59.9	28.4	11.8
VPOC_5_	62.8	24.5	12.7

### Surface acidity and catalytic performance

**Fig. 8:**
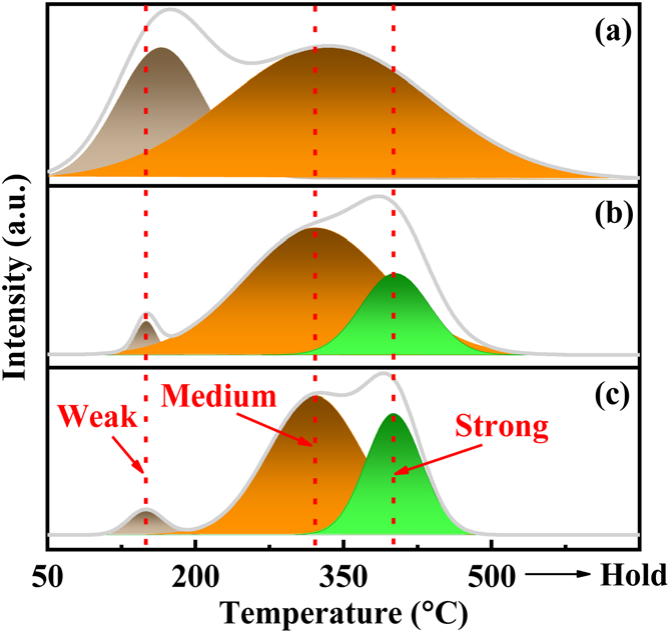
NH_3_-TPD profiles of the catalysts, (a) VPO, (b) VPOC_6_, (c) VPOC_5_.

The target reaction is a typical acid-catalyzed dehydration reaction. Therefore, the analysis of surface acidity *via* NH_3_-TPD plays a crucial role in investigating the structure–activity correlation of the catalyst. As depicted in Fig. 8, the NH_3_ desorption curve exhibits three distinct peaks corresponding to weak acid sites (140–170 °C), medium acid sites (320–350 °C), and strong acid sites (400–430 °C). The calibrated NH_3_ desorption amounts are listed in Table S4.† Evidently, the amorphous catalysts VPOC_5_ and VPOC_6_ exhibit significantly higher density of surface acidic sites compared to the crystalline catalyst VPO. The disparity can be attributed to the increased exposure of acidic sites resulting from the departure of alkaline amino compounds during the activation process. Furthermore, the catalyst VPOC_6_ prepared with 1,6-diaminohexane exhibits the highest surface acid site density and catalytic activity due to increased acidity resulting from the presence of variable V species along with abundant O_V_ and numerous hydroxyl groups,^39^ thereby enhancing the selectivity towards the desired product.

**Fig. 9:**
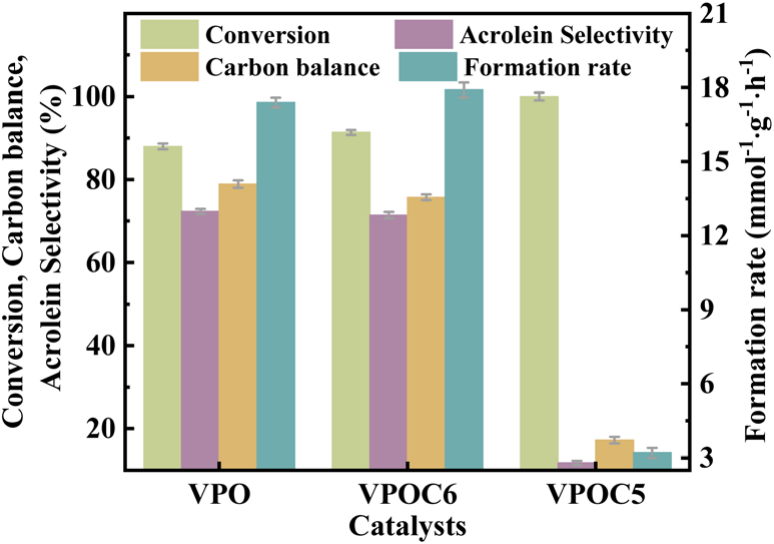
Catalytic performance of the catalysts, the reaction temperature and carrier flow rate was 320 °C and 30 mL per min (N_2_), respectively. The liquid feed was a glycerol aqueous solution (20 wt%), with a LHSV of 6 mL h^−1^.

The catalytic performance of the catalysts was evaluated in the glycerol dehydration reaction to acrolein under atmospheric pressure. The detailed evaluation procedures are detailed in the ESI. As shown in Fig. 9, the catalyst VPOC_5_ shows the highest glycerol conversion, accompanied by extremely low acrolein selectivity and carbon balance. It can be attributed to the action of *N*-methylpiperazine, which completely disrupts the vanadium–phosphorus oxide structure responsible for the selectivity of acrolein (as indicated by Raman results). Thus, although more acidic sites are formed on the catalyst surface, almost all converted glycerol is transformed into carbon deposits (as shown in Table 2). On the contrary, catalyst VPOC_6_ shows comparable selectivity for acrolein as compared to the catalyst VPO that prepared without complexant. However, the conversion and acrolein formation rate are significantly enhanced, reaching 91.4% and 17.9 mmol g^−1^ h^−1^, respectively, owing to the presence of abundant medium acid sites.

**Table 2:** Catalytic performance of the catalysts^a^

Catalysts	Selectivity (%)				
	Acrolein	Acrylic acid	Acetic acid	CO_*x*_	Others
VPO	72.3 ± 0.6	0.2 ± 0.01	3.4 ± 0.02	3.8 ± 0.02	20.3 ± 0.2
VPNC_6_	71.4 ± 0.6	0.5 ± 0.03	0.9 ± 0.01	2.7 ± 0.01	24.5 ± 0.3
VPNC_5_	11.8 ± 0.5	0.0 ± 0.01	4.5 ± 0.02	7.0 ± 0.03	76.7 ± 0.4

a The reaction temperature and carrier flow rate were 320 °C and 30 mL per min (N_2_), respectively. The liquid feed was a glycerol aqueous solution (20 wt%), with a LHSV of 6 mL h^−1^.

By integrating characterization results of Raman, XPS, H_2_-TPR, SEM, BET, and NH_3_-TPD, a reliable structure–activity correlation could be established. During the preparation of catalysts, the addition and pyrolysis of 1,6-diaminohexane induces a transition from crystalline to amorphous state while preserving the fundamental vanadium–phosphorus oxide phases. The amorphous structure is enriched with abundant oxygen vacancies and active graphite-type nitrogen on the catalyst surface, resulting in a plethora of medium acid sites that facilitate the target reaction. Furthermore, the formation of a porous honeycomb structure during this transition also contributes to enhancing the catalyst activity.

The dehydration mechanism of glycerol over solid acid catalysts has been systematically elucidated in previous studies.^40^ The catalytic process involves four distinct stages, the reaction is initiated by glycerol adsorption through C_α_–OH groups onto catalytic active sites. Subsequently, concerted transfer of C_α_–H and cleavage of the C_α_–O bond lead to the formation of ketene intermediates. These transient species undergo tautomerization to establish thermodynamically stable enol configurations. The final C–O bond scission accompanied by water elimination completes the acrolein formation. Kinetic analyses reveal that the initial chemisorption process and ketene intermediate generation constitute the rate-determining steps governing the overall reaction efficiency. Oxygen vacancies (O_V_) have been demonstrated to critically modulate catalytic performance through two primary mechanisms. First, these defects enhance surface acid site density *via* undercoordinated metal centers, significantly improving adsorption capacity for oxygenated substrates such as alcohols and carboxylic acids.^41^ Second, the O_V_ induce electronic redistribution at catalyst surfaces, specifically elevating the p-orbital energy states of adjacent oxygen atoms.^42^ This electronic perturbation enhances the nucleophilic character at active sites, thereby facilitating both α-hydrogen abstraction and ketene intermediate stabilization during the rate-limiting steps. In the current work, the amorphous VPOC_6_ catalyst with abundant surface oxygen vacancies demonstrated a significant increase in medium acid site density. This enhancement notably promoted both the initial chemisorption process and ketene intermediate generation, ultimately resulting in superior catalytic performance.

**Fig. 10:**
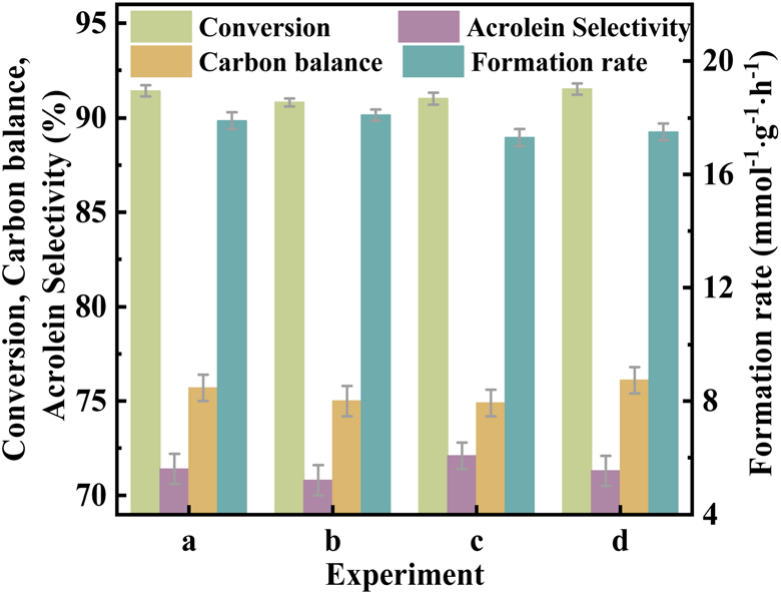
Catalytic performance of the catalyst VPOC_6_, (a) carrier flow rate = 30 mL min^−1^, LHSV = 6 mL h^−1^, catalyst amount = 0.5 g (20–40 mesh); (b) carrier flow rate = 60 mL min^−1^, LHSV = 12 mL h^−1^, catalyst amount = 1.0 g (20–40 mesh); (c) carrier flow rate = 90 mL min^−1^, LHSV = 9 mL h^−1^, catalyst amount = 1.5 g (20–40 mesh); (d) carrier flow rate = 30 mL min^−1^, LHSV = 6 mL h^−1^, catalyst amount = 0.5 g (40–60 mesh).

To assess potential mass transfer limitations in the reaction system, we conducted parametric analyses by systematically varying operational parameters. As shown in Fig. 10, the catalytic efficiency exhibited remarkable stability under proportional adjustments of key parameters, (1) reactant feed rate (6–18 mL h^−1^), (2) carrier gas flow rate (30–90 mL min^−1^), and (3) catalyst amount (0.5–1.5 g). Significantly, modulation of particle size (40–60 mesh range) also failed to produce detectable performance changes. These findings conclusively demonstrate that the reaction kinetics are dominated by intrinsic chemical processes rather than external transport constraints. The observed insensitivity to mass transfer conditions can be attributed to the hierarchically porous honeycomb architecture of VPOC_6_ catalyst, which facilitates rapid molecular diffusion through interconnected macroporous channels.

The catalytic performance of the VPOC_6_ catalyst is investigated under varying reaction temperatures, carrier gas flow rates, and oxygen contents, as depicted in Fig. S3† and 11. As Fig. S3 and Table S5† shows, the glycerol conversion exhibits a proportional increase with the rise in reaction temperature from 280 to 340 °C. However, acrolein selectivity initially increases and then decreases, reaching a peak of 73.7% at 300 °C. Consequently, the acrolein formation rate also peaked at this temperature (18.4 mmol g^−1^ h^−1^), indicating its optimality for the reaction. Furthermore, based on the carbon balance analysis, it was observed that the decrease in acrolein selectivity at elevated temperatures primarily resulted from an augmentation in carbon deposition.

**Fig. 11:**
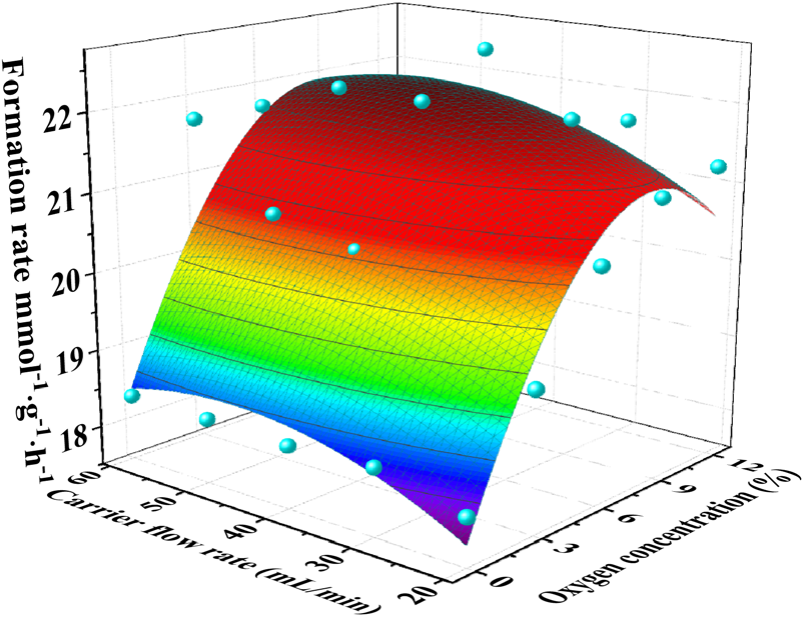
Catalyst performance of the catalyst VPOC_6_, the reaction temperature was 300 °C. The liquid feed was a glycerol aqueous solution (20 wt%), with a LHSV of 6 mL h^−1^.

The effects of carrier gas composition and flow rate on the catalytic performance of catalyst VPOC_6_ were systematically investigated, as shown in Fig. 11 and Table S6.† The presence or absence of oxygen in the carrier gas was found to have a significant impact on the production of acrolein, with distinct trends observed for the conversion and selectivity. Specifically, the flow rate of the carrier gas had a negligible influence on the acrolein formation rate in the absence of oxygen. However, as the flow rate increased, the conversion decreased while the selectivity increased. It can be attributed to the reduced contact time between the reactants and catalyst at higher flow rates, resulting in lower conversion and higher selectivity due to decreased deep product combustion. However, the acrolein formation rate initially increased and then decreased with increasing oxygen content. It because that an optimal oxygen content effectively regulates the vanadium oxidation state on the catalyst surface, thereby enhancing the surface acidity and catalytic activity. Through surface fitting analysis of experimental data, it was determined that highest acrolein formation occurred at an oxygen content of 8.6% in the carrier gas, with a flow rate of 47.4 mL min^−1^, indicating optimal catalytic performance under these conditions. Furthermore, the experimental results also demonstrated a reduction in carbon loss as the oxygen content increased. It is likely due to the heightened oxidation state of vanadium on the catalyst surface, which effectively inhibits carbon formation. It provides valuable insights for optimizing catalytic reaction conditions and improving overall catalytic performance.

### Catalyst durability

**Fig. 12:**
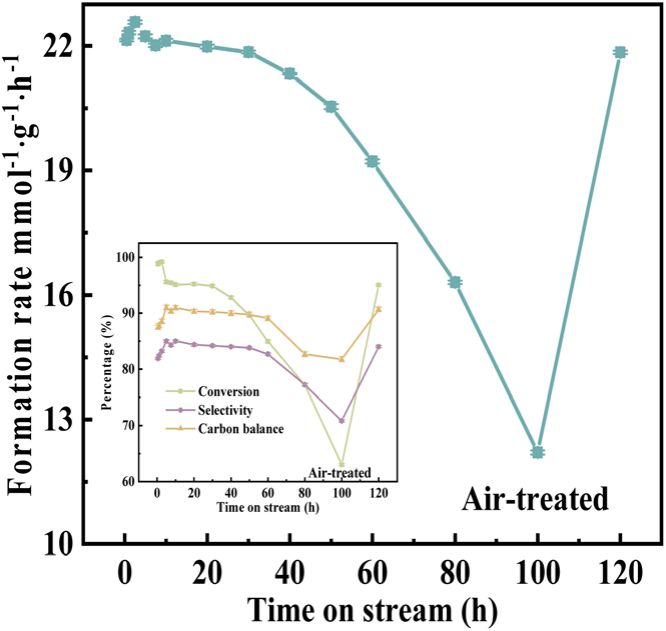
Durability test over catalyst VPOC_6_, the reaction temperature and carrier flow rate were 320 °C and 40 mL min^−1^ (9%-O_2_/N_2_), respectively. The liquid feed was a glycerol aqueous solution (20 wt%), with a LHSV of 6 mL h^−1^.

Under the optimal reaction conditions, durability testing was conducted on catalyst VPOC_6_, and the results were presented in Fig. 12. During the initial stage (2.5–5 h), a significant decrease in the rate of target product formation was observed, which then stabilized for 30 h, indicating an induction process of the catalyst during the initial stage. Subsequently, after 30 h, there was a gradual decline in the rate of target product formation, suggesting an accelerated deactivation process of the catalyst. However, as depicted in the inset of Fig. 12, glycerol conversion started to decline significantly at 30 h while acrolein selectivity remained stable until 60 h, thus, implying that conversion might be the primary factor contributing to the decline in formation rate.

The deactivated VPOC_6_ catalyst (after 100 h continuous operation) was regenerated through programmed thermal treatment in flowing air (60 mL min^−1^) with a controlled thermal protocol, (1) linear heating at 2 °C min^−1^ to 400 °C and (2) 16 h isothermal treatment. Post-regeneration catalytic testing (Fig. 12) demonstrated near-complete recovery of initial activity, confirming exceptional regenerability of the catalyst system. To assess structural stability, ICP-OES analysis tracked vanadium leaching dynamics during prolonged operation (Fig. S4†). The vanadium leaching revealed two distinct mechanistic phases. Within the initial 0.5 h, a pronounced leaching peak (147.5 mg L^−1^) emerged, attributed to the rapid dissolution of weakly adsorbed surface vanadium species. Significantly, catalytic activity remained unaffected during this period, demonstrating these surface-bound species are non-essential to active sites. Leaching rates decreased sharply to 20.1 mg L^−1^ by 1 h and stabilized at 5.7 mg L^−1^ from 5 h onward. This sustained leaching phase (5–100 h) exhibited a strong correlation with gradual activity decay (Fig. 12), suggesting progressive erosion of structurally integrated vanadium critical for catalytic cycles. Notably, regenerated catalysts displayed renewed leaching (5.8 mg L^−1^), which implies oxidative regeneration partially re-exposed active vanadium centers while preserving structural integrity. Thus, the discrepancy between leaching and activity is resolved by distinguishing two process, (1) transient surface leaching (0–5 h), involving non-active peripheral species with no performance impact, and (2) structural leaching (≥5 h), driven by degradation of the catalyst that directly governs deactivation.

Under comparable reaction conditions, the catalytic performance of amorphous VPOC_6_ for glycerol dehydration was systematically evaluated in comparison with crystalline catalysts (Table 3). While crystalline catalysts (AlP, W-KIT-6, ZSM-11, and 25% W-cSiO_2_) demonstrate superior initial activity and glycerol conversion attributable to their well-defined active site geometry and thermodynamic stability, their acrolein formation rates are significantly lower than that of VPOC_6_. This disparity arises from the limited density of accessible active sites on crystalline surfaces, which are constrained by rigid lattice structures, thereby hindering high-throughput reaction dynamics. In contrast, the amorphous VPOC_6_ catalyst features a porous honeycomb-like architecture with more active sites. The structural advantages synergistically contribute to VPOC_6_'s excellent durability (100 h), outperforming crystalline catalysts that typically deactivate within 6–15 h due to pore blockage or phase segregation. The amorphous framework also facilitates dynamic stabilization of V^3+^–V^4+^–V^5+^ redox couples (Fig. S5†), further improving acrolein selectivity.

**Table 3:** Literature data for the gas-phase conversion of glycerol over the acid catalysts

Reference	Catalysts	Conversion (%)	Selectivity (%)	Formation rate (mmol^−1^ g^−1^ h^−1^)	Durability (h)	Medium acid sites (μmol NH_3_ per g_cat_)
43	AlP	96	82	4.1	10	210
44	W-KIT-6	98	55	4.43	6	211
45	ZSM-11	92.2	68.1	0.81	9	97
3	25% W-cSiO_2_	97	54	4.22	12	161
Current	VPOC_6_	99.1	83.2	17.87	100	281.2

Systematic investigations have established that the surface density of medium acid sites fundamentally governs catalytic activity.^43^ As quantitatively demonstrated in Table 3, the amorphous catalyst VPOC_6_ exhibit significantly higher surface concentrations of medium acid sites than the crystalline catalysts, which directly correlates with superior performance in the target reaction. Notably, in metal phosphide systems, increased amorphous phase content promotes uniform acid site distribution across catalyst surfaces.^46^ Furthermore, the oxygen vacancy-rich nature of amorphous catalysts induces electronic restructuring, effectively enhancing both the nucleophilicity of active centers and intermediate formation kinetics.^42^ These combined factors-optimized acid site density and oxygen vacancy abundance-collectively account for the enhanced catalytic efficacy observed in amorphous catalyst relative to crystalline catalysts.

## Conclusions

In this study, amorphous V–P–N–C catalysts were prepared utilizing complexants, which are subsequently employed for catalyzing the dehydration of glycerol to produce acrolein. Compared to traditional crystalline vanadium–phosphorus oxide catalysts, amorphous V–P–N–C catalysts synthesized with 1,6-diaminohexane exhibited more pronounced graphitized nitrogen structure characteristics and demonstrated higher catalytic activity. It should be noted that the types of complexant plays a crucial role in determining both the density of acid sites and the catalytic performance. Through a comprehensive analysis that encompassed XRD, Raman, SEM, BET, H_2_-TPR, XPS and NH_3_-TPD, the mechanism of 1,6-diaminohexane in the catalyst preparation process could be elucidated. The integrity of the vanadium–phosphorus oxide phases was maintained, while the complexant facilitated the transition from crystalline state to amorphous state, resulting in the formation of an active graphitized nitrogen structure. The resulting amorphous structure imparts a multitude of O_V_ and variable V species on the surface. Consequently, the structure generates a significant quantity of medium acidic sites, which demonstrate exceptional catalytic activity toward target reactions. Additionally, the transition from crystalline to amorphous state also facilitates the formation of porous honeycomb structures, thereby significantly enhancing the catalytic activity.

## Data availability

The data supporting this article have been included as part of the ESI.†

## Author contributions

Jun Liu, Xinzhen Feng and Weijie Ji designed and conducted this research. Jun Liu, Xiaobing Zhao, Weichen Wang, and Youjun Yan analyzed data. Jun Liu and Xinzhen Feng wrote the paper. Weijie Ji modified the paper. Jun Liu, Guofu Huang, and Meng Liang plotted the figures. Jun Liu, Xinzhen Feng, and Weijie Ji edited the whole manuscript.

## Conflicts of interest

The authors declare no competing interests.

## Supplementary Material

RA-015-D4RA08613A-s001
