# Supplementary material for: Acrolein production from glycerol dehydration over amorphous V–P–N–C catalysts
Source: RSC Adv. 2025 Apr 1;15(13):9801–9. doi: 10.1039/d4ra08613a (PMC11959359; doi:10.1039/d4ra08613a)
Supplement: RA-015-D4RA08613A-s001 [file RA-015-D4RA08613A-s001.pdf]

Supplementary Information (SI) for RSC Advances.
This journal is © The Royal Society of Chemistry 2025

Supplementary Information

Highly efficient Amorphous V-P-N-C Catalysts for sustainable production of Acrolein through Glycerol Dehydration

Jun Liu,[a,b] Xiaobing Zhao,[a] Weichen Wang,[a] Youjun Yan,[a] Guofu Huang,[a] Meng Liang,[a] Xinzhen Feng,[*b] and Weijie Ji[b]

[a]Peninsula Engineering Research Center of Comprehensive Brine Utilization, Weifang University of Science and Technology, Weifang, 262700, China

[b]Key Laboratory of Mesoscopic Chemistry, MOE, School of Chemistry and Chemical Engineering, Nanjing University, Nanjing, 210023, China

[*]E-mail: fxz@nju.edu.cn

*Corresponding authors. Tel: +86-25-89686270, Fax: +86-25-89687761,
E-mail: fxz@nju.edu.cn (X.Z. Feng)

Characterization details

Raman. The Raman spectra were recorded at RT on a HORIBA LabRAM HR Evolution Raman spectrometer (laser source: 532 nm).

XRD. X-ray powder diffraction (XRD) patterns were recorded on a Philips X'Pert MPD Pro X-ray diffractometer with graphite monochromatized Cu Ka radiation (k = 0.1541 nm).

XPS. The binding energy (BE) was calibrated against the C1s signal (284.6 eV) of contaminant carbon. Elemental surface composition was estimated on the basis of peak areas normalized using Wagner factors. Relative surface concentration of C, N, V, and O element with different states can be estimated through deconvolution analysis of the corresponding XPS peak. For the same batch of sample measured under identical conditions as well as the same parameters adopted for deconvolution analysis.

H_2-TPR. Hydrogen temperature-programmed reduction (H_2-TPR) was performed from room temperature (RT) to 850 °C at a rate of 10 °C/min in a flow of 5% H_2/Ar (v/v, flow rate = 40 mL/min) and isothermally held at 850 °C until reduction was complete.

NH_3-TPD. Catalyst of 50 mg was first heated in an Ar flow (40 mL/min) to 200 °C and kept at this temperature for 1 h. Then the sample was cooled to 50 °C in the Ar flow. After that, NH_3 adsorption was performed at 50 °C for 1 h. Finally, NH_3-TPD was carried out in an Ar flow (40 mL/min) with the sample being heated to 500 °C at a rate of 10 °C/min. The amount of desorbed NH_3 (in μmol/g) was determined by a titration, in which a HCl solution (0.01 mol/L) was used to absorb the released NH_3. A NaOH solution (0.01 mol/L) was used as the titrant.

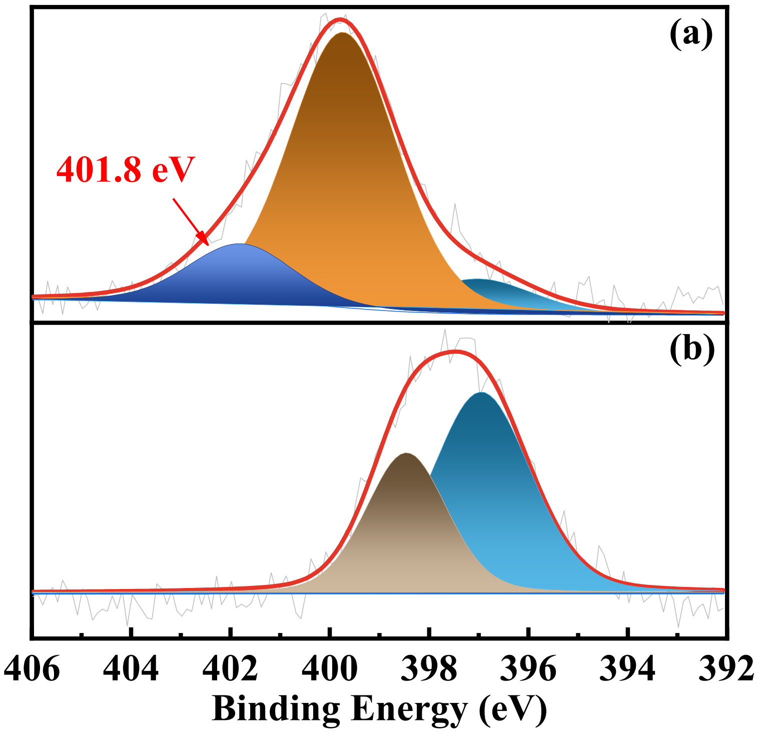

Fig. S1 XPS spectra of N 1s for the catalysts: (a) $VPOC_6$, (b) $VPOC_5$.

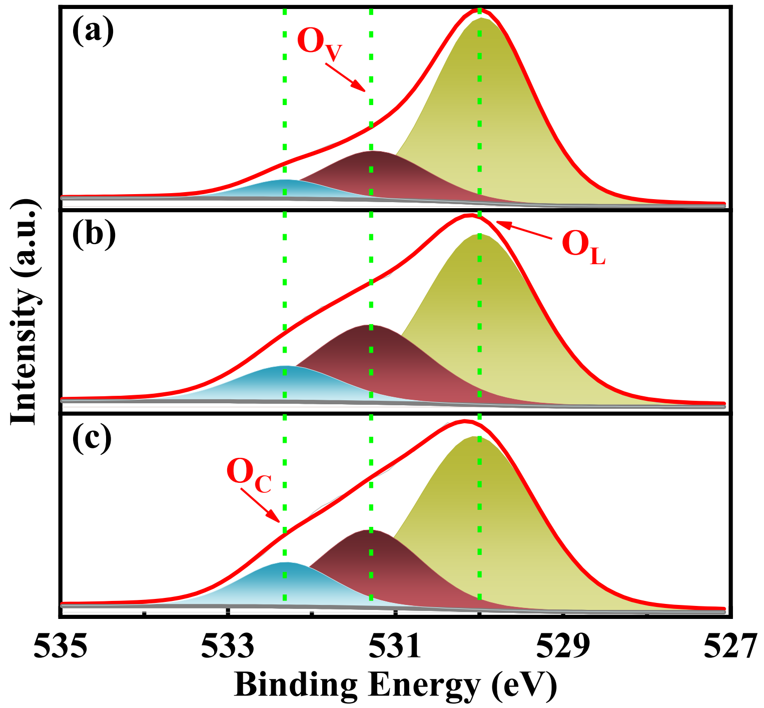

Fig. S2 XPS spectra of O 1s for the catalysts: (a) VPO, (b) $VPOC_6$, (c) $VPOC_5$.

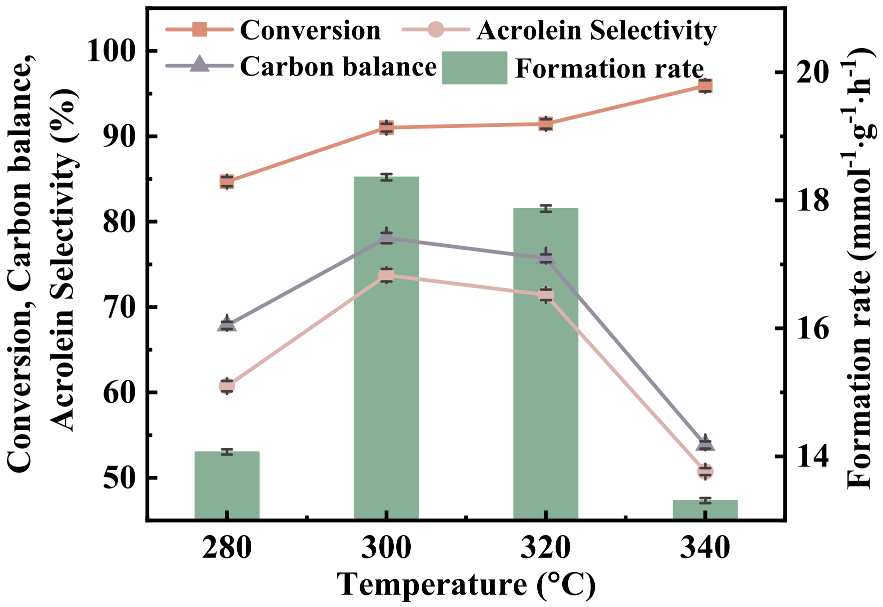

Fig. S3 Catalyst performance of the catalyst $VPOC_6$, the carrier flow rate was 30 mL·min^{-1} (N_2). The liquid feed was a glycerol aqueous solution (20 wt%), with a LHSV of 6 mL·h^{-1}.

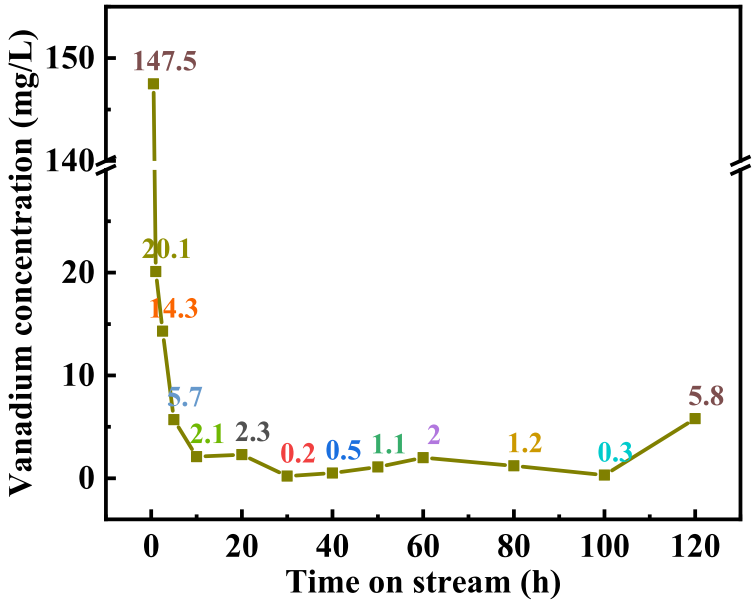

Fig. S4 Leaching test over catalyst $VPOC_6$, the reaction temperature and carrier flow rate was 320°C and 40 mL·min^{-1} (9%-O_2/N_2), respectively. The liquid feed was a glycerol aqueous solution (20 wt%), with a LHSV of 6 mL·h^{-1}.

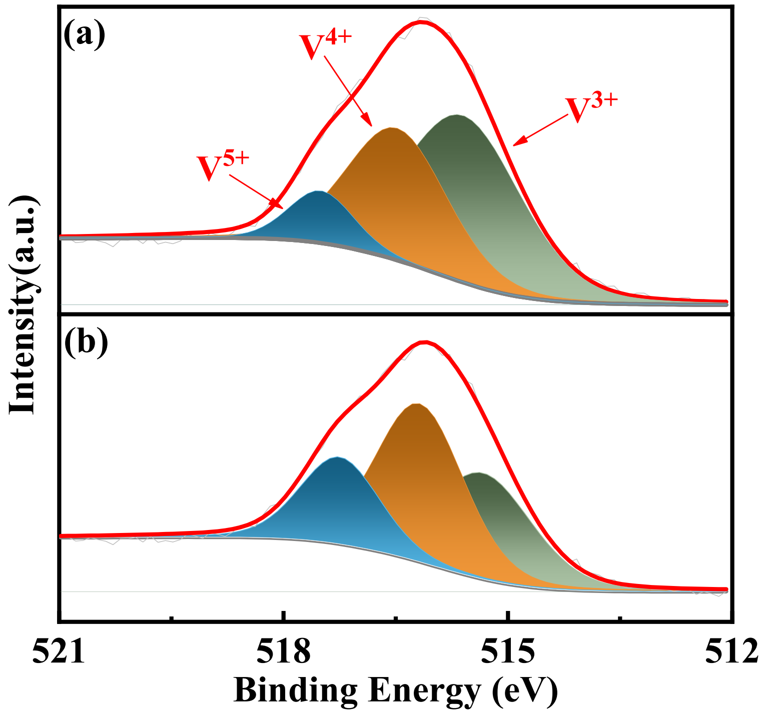

Fig. S5 XPS spectra of V $2p_{3/2}$ for the catalysts, (a) fresh $VPOC_6$, (b) $VPOC_6$, utilized 60 hours.

Table S1 Textural properties of the catalysts.

Catalysts	Surface area (m^2/g)	Pore volume ($\times10^{-3}$ cm^3/g)	Average Pore (nm)
**VPO**	6.00	40.90	31.45
**$VPNC_6$**	3.35	17.40	20.33
**$VPNC_5$**	2.50	12.25	16.73

Table S2 H_2-TPR profiles of the catalysts.

Catalysts	V^{5+} (°C)		V^{4+} (°C)		Ratio of V^{5+}/V^{4+}
	Temp. (°C)	H_2 Consumption (mmol/mol V)	Temp. (°C)	H_2 Consumption (mmol/mol V)	
**VPO**	594	6.92	804	23.07	0.30
**VPOC6**	451	2.25	833	8.03	0.28
**VPOC5**	528	2.38	833	9.92	0.24

Table S3 XPS results of V 2p for the catalysts.

Catalysts	V^{5+} (%)	V^{4+} (%)	V^{3+} (%)
**VPO**	62.6	37.4	/
**VPNC6**	9.4	36.7	53.9
**VPNC5**	/	55.0	45.0

Table S4 The surface acidity of the catalysts.

Catalysts	Acid site distribution (μmol NH_3/g_{cat})			Total acidity (μmol NH_3/g_{cat})
	Weak	Medium	Strong	
**VPO**	53.2	128.1	/	181.3
**VPNC6**	10.9	281.2	86.0	378.1
**VPNC5**	12.5	183.3	94.7	290.5

Table S5 Catalyst performance of the catalyst $VPOC_6$.

Temperature (°C)	Selectivity (%)				
	Acrolein	Acrylic Acid	Acetic Acid	CO_x	Others
**280**	60.7±0.6	0.4±0.02	0.4±0.02	2.1±0.02	36.4±0.2
**300**	73.7±0.7	0.8±0.03	1.1±0.02	2.1±0.03	22.3±0.2
**320**	71.4±0.6	0.5±0.03	0.9±0.01	2.7±0.01	24.5±0.3
**340**	50.7±0.4	0.3±0.02	0.4±0.01	1.6±0.02	46.9±0.4

* The carrier flow rate was 30 mL·min^{-1} (N_2). The liquid feed was a glycerol aqueous solution (20 wt%), with a LHSV of 6 mL·h^{-1}.

Table S6 Effect of oxygen concentration and carrier flow rate.

carrier flow rate (mL)	oxygen concentration (%)	Conversion (%)	Selectivity (%)					Carbon Balance (%)
			Acrolein	Acrylic Acid	Acetic Acid	CO_x	Others	
20	0	92.3±0.5	71.7±0.4	0.7±0.03	2.6±0.04	2.1±0.02	22.9±0.1	76.8±0.7
	3	93.4±0.4	75.4±0.4	0.9±0.04	0.5±0.02	2.6±0.03	20.6±0.1	79.0±0.7
	6	95.4±0.4	78.4±0.4	1.1±0.03	0.3±0.03	2.7±0.03	17.5±0.1	81.5±0.4
	9	96.1±0.3	80.1±0.6	1.0±0.03	0.4±0.03	4.4±0.04	14.0±0.1	83.5±0.5
	12	98.3±0.4	79.0±0.5	2.2±0.04	1.4±0.04	7.6±0.04	9.7±0.1	85.0±0.5
30	0	91.0±0.3	73.7±0.7	0.8±0.03	1.1±0.02	2.1±0.03	22.3±0.2	78.1±0.3
	3	94.5±0.5	76.7±0.7	0.9±0.02	0.4±0.02	2.6±0.04	19.4±0.1	80.0±0.2
	6	96.0±0.2	79.5±0.2	1.3±0.04	0.2±0.01	4.3±0.03	14.6±0.2	83.1±0.2
	9	98.6±0.2	81.0±0.3	2.0±0.03	0.3±0.02	7.8±0.04	9.0±0.1	85.9±0.3
	12	99.4±0.2	79.6±0.2	3.4±0.04	1.0±0.03	13.2±0.03	2.7±0.05	88.2±0.2
40	0	89.8±0.7	74.5±0.4	0.9±0.02	4.7±0.07	2.0±0.03	17.9±0.1	81.3±0.4
	3	95.3±0.5	78.4±0.5	1.0±0.03	1.1±0.03	2.8±0.04	16.6±0.2	82.0±0.5
	6	98.0±0.3	82.3±0.2	0.5±0.02	0.9±0.03	7.7±0.03	8.5±0.1	86.3±0.6
	9	99.1±0.2	83.2±0.2	0.6±0.03	0.4±0.02	13.0±0.03	2.8±0.05	88.5±0.4
	12	100±0.2	78.2±0.5	4.8±0.04	0.6±0.03	15.9±0.04	0.5±0.03	88.7±0.4
50	0	90.1±0.7	74.3±0.4	0.8±0.02	10.4±0.1	2.3±0.02	12.1±0.1	84.6±0.3
	3	96.0±0.7	78.8±0.7	1.1±0.03	6.6±0.08	4.1±0.03	9.4±0.1	86.2±0.2
	6	98.6±0.2	81.9±0.2	1.2±0.04	3.0±0.04	8.2±0.03	5.7±0.1	88.0±0.2
	9	100.0±0.2	80.2±0.3	1.0±0.03	4.4±0.03	14.3±0.04	0.04±0.01	88.9±0.2
	12	100.0±0.2	77.0±0.7	6.2±0.05	1.3±0.02	15.4±0.05	0.05±0.01	89.2±0.3
60	0	88.3±0.8	75.9±0.8	1.0±0.03	9.4±0.1	2.2±0.03	11.6±0.1	85.7±0.5
	3	98.0±0.4	81.0±0.2	1.1±0.03	3.7±0.03	4.4±0.04	9.8±0.1	86.3±0.4
	6	99.1±0.2	80.0±0.2	1.0±0.03	4.9±0.04	13.3±0.05	0.8±0.01	88.8±0.2
	9	100.0±0.2	77.6±0.8	4.0±0.03	4.4±0.03	13.9±0.06	0.2±0.01	89.1±0.2
	12	100.0±0.2	76.4±0.7	6.8±0.04	2.3±0.03	14.2±0.05	0.4±0.02	89.4±0.2

* The reaction temperature was 300 °C. The liquid feed was a glycerol aqueous solution (20 wt%), with a LHSV of 6 $mL\cdot h^{-1}$.
